# Algorithmic harms and digital ageism in the use of surveillance technologies in nursing homes

**DOI:** 10.3389/fsoc.2022.957246

**Published:** 2022-09-16

**Authors:** Clara Berridge, Alisa Grigorovich

**Affiliations:** ^1^School of Social Work, University of Washington, Seattle, WA, United States; ^2^Recreation and Leisure Studies, Brock University, St. Catharines, ON, Canada

**Keywords:** artificial intelligence, technology, dementia, big data, older adults, long-term care, machine learning, privacy

## Abstract

Ageism has not been centered in scholarship on AI or algorithmic harms despite the ways in which older adults are both digitally marginalized and positioned as targets for surveillance technology and risk mitigation. In this translation paper, we put gerontology into conversation with scholarship on information and data technologies within critical disability, race, and feminist studies and explore algorithmic harms of surveillance technologies on older adults and care workers within nursing homes in the United States and Canada. We start by identifying the limitations of emerging scholarship and public discourse on “digital ageism” that is occupied with the inclusion and representation of older adults in AI or machine learning at the expense of more pressing questions. Focusing on the investment in these technologies in the context of COVID-19 in nursing homes, we draw from critical scholarship on information and data technologies to deeply understand how ageism is implicated in the systemic harms experienced by residents and workers when surveillance technologies are positioned as solutions. We then suggest generative pathways and point to various possible research agendas that could illuminate emergent algorithmic harms and their animating force within nursing homes. In the tradition of critical gerontology, ours is a project of bringing insights from gerontology and age studies to bear on broader work on automation and algorithmic decision-making systems for marginalized groups, and to bring that work to bear on gerontology. This paper illustrates specific ways in which important insights from critical race, disability and feminist studies helps us draw out the power of ageism as a rhetorical and analytical tool. We demonstrate why such engagement is necessary to realize gerontology's capacity to contribute to timely discourse on algorithmic harms and to elevate the issue of ageism for serious engagement across fields concerned with social and economic justice. We begin with nursing homes because they are an understudied, yet socially significant and timely setting in which to understand algorithmic harms. We hope this will contribute to broader efforts to understand and redress harms across sectors and marginalized collectives.

## Introduction

Surveillance technologies are adopted within nursing homes to remotely monitor older adults and workers with an interest in improving quality of care, health, and safety (Niemeijer et al., 2015; Vermeer et al., 2019; Mitchell et al., 2020; Lukkien et al., 2021; World Health Organization, 2022). Vermeer et al. (2019) define surveillance technology as comprising “monitoring systems that can allow for 24-h supervision by caregivers” (p. 2). In the context of COVID-19, there has been a push to adopt or repurpose these technologies to assist with digital contact tracing or to support infection and control practices (NAS, 2022). Examples include Real Time Locating Systems (RTLS), fall detection systems, activity sensors, and tracking apps (Grigorovich and Kontos, 2020; Chandonnet, 2021; Fan et al., 2021; Grigorovich et al., 2021). Increasingly, these devices employ algorithms to automate recognition of certain deviations in activities or movements and to trigger responses to pre-programmed parameters (e.g., alert triggered if resident spends too long in one spot as compared to their routine; resident approaches a virtual fence). Such technologies may also involve the application of natural language processing (chatbots, and social robots), or machine learning techniques to process continuously collected information about the movements, location, activities, and physiological state of older adults in their environment (Chandonnet, 2021; Orlov, 2021). There is also increasing interest in expanding the role of artificial intelligence (AI) in nursing homes to process information collected by a full range of technologies, including CCTV cameras, virtual assistants, and electronic health records, and to develop predictive algorithms and automated decision-making systems about care (Wojtusiak et al., 2021; Khan et al., 2022; Zhu et al., 2022). The push to adopt surveillance technologies in nursing homes is thus largely driven by their imagined future benefit for prevention and quality improvement through more timely identification of adverse events and personalization of care in the context of widespread staffing shortages. There has been limited attention to the potential harms of reliance on these technologies to older adults, workers, and others, or on the ways in which they may contribute to ageism.

In this paper, we describe the need for greater attention by gerontology and age studies scholars to bodies of work on algorithmic harms within critical scholarship on information and data technologies where ageism is not yet addressed. The aim of this paper is to bridge this disciplinary gap. Our guiding question in this exploration is, how would engagement with critical data and information scholarship direct critical gerontology research concerned with algorithmically-mediated decision making and AI in nursing homes?

We focus on surveillance technologies in the nursing home context because it has been made politically urgent by COVID-19 and is a generative inroad to deepening analyses of what has been termed “digital ageism” (Manor and Herscovici, 2021; Chu et al., 2022). We use the terms AI and algorithmic as shorthand to reflect the terms popularly used, which for the purpose of this paper reference an assemblage of technologies that support algorithmically mediated decision making, including technologies like sensors that are not properly AI but that will create the data for machine learning and automated decision making in the future. We discuss harms as systemic discrimination resulting from the use of surveillance technologies to enhance the collection and production of “actionable intelligence or guidance…that result in the reinforcement or exacerbation of inequality within society” (Gandy, 2010, p. 3). Such decisions are based on value-laden classifications that can entrench unequal relationships. Examples of algorithmic harms include wrongful arrests, employment exclusion, unfair allocation of publicly funded care, reputational harm, misrecognition, and financial, emotional, and psychological harms (Redden and Brand, 2020; Moss et al., 2021; Malik et al., 2022). Harms have been categorized by level at which they occur, including individual harm, community harm, and social harm (Smuha, 2021). Identification of algorithmic harms is ongoing (Redden and Brand, 2020), and taxonomies are expanding as they include “an ever-widening circle of consequences” to individuals, their families, and other connected individuals (Moss et al., 2021, p. 6). Algorithmic harms can be intentional and unintentional (Redden and Brand, 2020).

We do not offer a taxonomy of technologies or algorithmic harms in nursing homes because more research is needed to document the specific applications of AI in nursing homes and their implications. While there are a significant number of surveillance technologies marketed to nursing homes, we still lack a clear picture of how these are incorporated at the organizational level. This inattention to what is currently used in facilities is part of the problem. To begin to map future directions for research on algorithmic harms, we draw insights from the body of research on surveillance technologies in elder care that are incorporated in algorithmically mediated decision making and the limited work on their use within nursing homes.

### Nursing home context

The nursing home is a central challenging site for gerontology and age studies. Widely considered an undesirable place to reside, nursing homes are the pivotal event horizon in conceptualizations of the fourth age, existing in the social imaginary as a form of social death and casting a foreboding shadow on older adults striving to achieve third age functionality (Higgs and Gilleard, 2014). Yet a sizable minority of people residing in nursing homes are “low-care residents” who are considered able to live in a less restrictive environment (Mor et al., 2007; Thomas and Mor, 2013). About two-thirds of U.S. nursing home residents are covered by Medicaid, and states are required to provide nursing home care but not home and community-based services under Medicaid. In Canada, nursing and personal care services in nursing homes are publicly funded, but residents typically pay means-tested monthly accommodation payments, and other types of supported living alternatives like retirement homes or memory homes for people living with dementia are paid for entirely out of pocket. This institutional bias contributes to the high waiting lists for state-funded in-home long-term services and supports (Grabowski, 2021). Less institutional options like assisted living and continuing care retirement communities remain financially and locationally inaccessible to many, particularly poor and Black older adults (Jenkins Morales and Robert, 2020; Sloane et al., 2021).

In the U.S., nursing homes that can admit private-pay residents do so to avoid Medicaid's lower reimbursement rates (Sloane et al., 2021), and they remain highly racially segregated despite recent reductions in the proportion of white residents and significant increases in Black and Hispanic residents (Feng et al., 2011). Both Black and Hispanic nursing home residents are more likely than white residents to live in facilities with low staffing ratios, high inspection problems, lower revenue (% Medicaid & occupancy), and in those that may be slated to be closed (Mor et al., 2004; Smith et al., 2008; Fennell et al., 2010; Li et al., 2015). Largely due to their segregation into lower quality facilities, Black nursing home residents as compared with their white counterparts report lower quality of life (Shippee et al., 2020), are more often physically restrained (Cassie and Cassie, 2013), and receive pain treatment less often (Mack et al., 2018). While race, ethnicity, and Indigeneity information is not available for Canadian nursing homes, there is some evidence of similar trends; for example, people wait longer for placement for a basic bed (vs. a private one), and those for whom neither English nor French is their first language experience longer wait times (Flanagan et al., 2021).

Most of the direct care of nursing home residents in the U.S. and Canada is provided by immigrant and racially marginalized women, specifically Black, Latina, and Asian American and Pacific Islander women (Wagner et al., 2021). These workers are often poorly paid (Estabrooks et al., 2020; Scales, 2022), insecurely employed, and not unionized (Sojourner et al., 2010; Zagrodney and Saks, 2017). Many leave this work, unable to envision life-long careers there (Brannon et al., 2007). Nursing home residents tend to be very old (more than half in Canada are over 85 years old), disabled, and living with chronic conditions; however, a sizable minority−17% and 7% in the U.S. and Canada, respectively—are younger than 65 (U. S. Census Bureau, 2018; OLTCA, 2019). But unlike other movements to deinstitutionalize disabled people, no large-scale abolition movement for nursing homes has developed, nor have gerontologists or resident advocates forcefully called for one (Herron et al., 2021). The nursing home plays an outsized role in the social imaginary and in ideas of what old age entails, and yet it is a site that seems to impoverish our imagination for how it could be otherwise.

### “Digital ageism” and the digital push response to COVID-19

COVID-19 is said to have created a “perfect storm” in the crisis of nursing homes infection spread and deaths (Konetzka et al., 2021). Just prior to the pandemic, U.S. domain experts identified a range of specific ongoing problems in the industry and concluded that “the current widespread violations of international covenants and conventions and domestic laws and regulations should be considered a national emergency, and government plans should be put in place to address the urgent human rights crisis in nursing home care” (Harrington et al., 2019, p. 68). The COVID-19 crisis drew public attention to myriad problems within nursing homes, while at the same time further entrenching them as a site of failure, social abandonment, and isolation. About one third of COVID-19 fatalities in the U.S. have occurred in long-term care facilities—the vast majority nursing homes—while less than 0.5% of the U.S. population live in nursing homes (The New York Times, 2021). In Canada, nursing home residents account for less than 1% of the population but 43% of COVID-19 deaths (CIHI, 2021a). Numerous op-eds and policy research have highlighted inadequate staffing, regulatory and infection control problems, and vulnerabilities inherent in congregate living (Armstrong et al., 2020; Werner et al., 2020). But public attention quickly shifted away from demand for structural change and permanent policy responses are still forthcoming.

A response that has not faded is the “digital push” in nursing homes (Gallistl et al., 2021). The ways in which COVID-19 laid bare inadequate connective technology infrastructure in this setting has triggered a renewed call to address the digital divide. For example, incorporation of Electronic Health Records in U.S. nursing homes lagged considerably behind beneficial use in other sites of healthcare, leaving some residents with incomplete and inaccessible paper records, with negative implications for person-centered and quality care (NAS, 2022). Certainly, the digitally disconnected residential life behind closed nursing home doors where internet or connective devices were inadequate exposed the harmful, isolating reality of the ageism and ableism embedded in this digital divide.

While the digital divide has always been central to the subfield of gerontechnology (Czaja et al., 2001; Charness and Boot, 2009; Rogers and Fisk, 2010), a concept that has emerged and is gaining traction as an outgrowth of it is “digital ageism” (Chu et al., 2022). Chu et al. (2022) define digital ageism as “age bias in technology such as AI” (para 2). Ageism is often conceptualized as negative attitudes and discrimination based on prejudices about age (Butler, 1969; FrameWorks Institute, 2017). The analytics and dominant discourses of ageism have been critiqued for centering whiteness (Herron et al., 2021), a problem that digital ageism scholarship risks reinforcing. Where AI is concerned, ageism is presented as bias and exclusion (from tech design considerations, for example) based on stereotypes (Manor and Herscovici, 2021). Whether the focus is on lack of access or other forms of exclusion, the predominant concern has been the extent to which digital ageism, such as that embedded within the youth-focused tech and design industries, negatively affects the use of technologies by older adults (see Manor and Herscovici, 2021). Ageist exclusion has also been identified in limited or stereotypical representation of old age or older adults themselves within data training sets (Diaz et al., 2018; Taati et al., 2019; Rosales and Fernández-Ardèvol, 2020; Chu et al., 2022). Researchers are thus making the case that concerns of ageism belong in the AI bias literature alongside racism, ableism, and sexism (Rosales and Fernández-Ardèvol, 2020; Stypińska, 2021; Chu et al., 2022). Stypińska (2021) has offered the broadest discussion of how ageism appears in AI, while noting the dearth of theoretical reflection and research on algorithmic harms for older people as a group, such as discriminatory decision making and stereotypically negative representation that entrenches ageism. We agree that the explicit application of the idea of ageism to digital technology, and specifically AI, is overdue. We too are interested in the rhetorical power of ageism, in addition to its analytical use, because to name a phenomenon is to be able to unveil, critique, and change it. Age should be examined alongside other socio-political categories in the AI bias literature, but we argue that the more pressing analysis takes us in a divergent direction from that literature.

We contend that a hard look at the algorithmic harms of surveillance technologies will deepen our understanding of ageism and reframe a research agenda for gerontology in the multigenerational service of older adults and direct care workers. We propose that fields of study and practice concerned with digital ageism (i.e., gerontechnology, age studies, gerontology) would benefit enormously from engaging with critical scholarship on disparate effects of automation and algorithmic decision-making systems that have been advancing, particularly in fields of critical race, feminist and disability studies and activism. In turn, incorporating analysis of ageism could prove generative for these fields. Nascent work on digital ageism and ageism in AI has focused on principles that align with other mainstream critiques of AI, namely resolving the problem that is largely defined as bias (in this case against older adults) by implementing fairness and inclusion (Hoffmann, 2021). That work seeks to address other biases like racial and gender bias in datasets that are linked to problems like unfair hiring, advertising, credit scoring, and risk assessing (Miceli et al., 2022). As Miceli et al. (2022) have argued, bias studies keep the problem within the realm of data and technology, which “obscures its root causes” and power asymmetries (p. 2). Inclusion promises to resolve the bias problem through and within information and data technologies. That is, data harms are said to be caused and resolved by the same technologies (Hoffmann, 2020b). In this way, expertise is allocated to the tech sphere, and the norms, social hierarchies, and asymmetrical vulnerabilities entrenched by the use of surveillance technologies, and AI specifically, are left unquestioned (Hoffmann, 2020b). For example, in her analysis of the ways in which poverty is managed through surveillance technologies, Eubanks (2018) has shown how inequality is automated through algorithmic decision making regarding public housing, Medicaid, and child welfare, with inordinate surveillance of those living in poverty. She thus argues that where “systems engineering approaches to social problems” are at play, impact must be front and center, regardless of intention.

Much of the research and current efforts to get aging on the radar of AI bias research is uninformed by critiques of ethics washing or the gold standard of fairness and inclusion that have been central to critiques of AI within aforementioned fields (Greene et al., 2019; Hoffmann, 2019; Green, 2021). Like the larger bias literature, the digital ageism concept is shaping up to offer critiques of bias and exclusion in AI and imply that the answer can be found in some combination of representations of old age and older adults that are deemed less biased to data training sets and in, essentially, tweaking algorithms. Desire for inclusion is taken as a given and paired tightly with calls to bridge the digital divide. The scope and nature of the problems introduced by AI are confined to AI and/or articulated on AI's terms, rather than understanding how the introduction of AI technologies reorganize subjects and relationships regardless of how nominally fair or inclusive they are. We argue that there are more elements of ageism at play that are obscured by calls for inclusion.

Critical scholarship on information and data technologies understands data technologies as a structuring force, which digital ageism discourse has not thus far articulated. Because data are never neutral or without ethical salience, digital ageism cannot be reduced to a consequence of “good” or “bad” design, training sets, or intended use. Our focus on the nursing home reveals how digital ageism discourse is not attending to the ways in which AI can retrench power hierarchies that are rooted in racism, ableism, sexism, and classism, foregrounding promise of imagined future benefit that forestalls critical engagements with structural, racist logics. These limitations are elucidated through engagement with critical scholarship on data technologies offered in fields that gerontology could be better informed by. We begin with an overview of insights from critical disability studies, critical race studies, and feminist studies. After presenting a limited overview of each, we discuss some of many potential paths of future study that are illuminated by this critical scholarship. We conclude by discussing the need to understand and articulate the social harms of AI in relation to the increasing adoption of surveillance technologies within nursing homes.

## Where can we look for critical insights on algorithmic harms?

### Critical disability studies

Disability studies scholars and activists have demonstrated how the introduction of automated algorithmic decision making in education, employment, and insurance promotes discrimination against embodied difference in ways of communicating, interacting, and being. For example, automated online proctoring has unfairly penalized people with disabilities through algorithms that flag some gestures and eye movements as “abnormal” or “unacceptable” exam behavior (Coghlan et al., 2021). Similarly, the use of HireVue and similar facial recognition technologies for automation of interview selection has been shown to discriminate against autistic and neurodivergent people for recommendations for job interviews (Whittaker et al., 2019). Large corporations employ automated decision making in hiring at least in part because it offers an efficient means for screening out those “deemed inefficient, non-productive, and likely to require extra help and support” (Whittaker et al., 2019). Such harms cannot be overcome through better or more data due to the diversity of disability and because the basic logic of AI is to find patterns and form groups to create simple models where outliers from the average are treated as “noise” and disregarded (Trewin, 2018; Whittaker et al., 2019).

Disability studies scholars have thus argued that efforts to reduce algorithmic harms must extend beyond the data collected and the technological design to interrogate “techno-ableism” that drives the development of AI and other technologies for detecting, curing, normalizing or modifying disability (Shew, 2020, p. 4). Connecting this to earlier critiques of medical and technological interventions on disabled lives (Davis, 2003; Garland-Thompson, 2006; Hamraie and Fritsch, 2019), these scholars demonstrate how AI technologies similarly rely on the assumption that we need to “overcome” disability through technology rather than address the structural causes of inaccessibility and discrimination experienced by disabled people. While AI and surveillance technologies are promoted as empowering or enhancing access for disabled people, these can further pathologize and retrench their unequal access to material goods. Moreover, they often amplify disabled peoples' exposure to invasive surveillance that requires intimate data sharing with for-profit companies. As an example, the growing interest in surveillance technologies and AI within health and rehabilitation sectors to “normalize, reprogram, or extinguish autistic traits” is a form “tech-saviorism” that reflects eugenic logics (Williams and Gilbert, 2020, p. 4). These technologies are used to classify and predict behavior for the purpose of coding, analysis, and interventions aimed at modifying the behaviors of autistic individuals (Williams, 2019; Williams and Gilbert, 2020). Rather than addressing the structural causes of “challenging behaviors,” including emotional or sensory distress from being forced to conform to neurotypical ideals, these technologies reproduce ableism by centering ability as the main goal of disability design and prioritizing the interests of caregivers and corporations (Shew, 2020).

### Critical race studies

Critical race studies scholars have demonstrated that deployment of automated algorithmic decision-making in social media, policing, education, social welfare, and employment re(produce) racial disparities in accuracy and impact for Black, Indigenous, and People of Color (BIPOC). They counter the assumption that these forms of racial bias are accidental, or merely a reflection of the dataset, by demonstrating that both the data and the technologies are built on assumptions of racial difference (that is, assumptions about race, risk, and value) that amplify racial hierarchies because racism is both desirable and profitable. For example, Noble (2018) has identified racial profiling by for-profit search engines that privilege whiteness and discriminates against women of color who are represented in erroneous, stereotypical and pornographic ways because this reflects the commercial interests that drive how information is categorized and analyzed. Similarly, a study by Obermeyer et al. (2019) showed that a widely used algorithm for selecting patients for access to healthcare programs was less likely to identify Black patients because it relied on the predictive utility of an individual's previous health expenses. This algorithm reproduced racism by using a metric that was insensitive to the structural reasons why Black patients had lower health care costs, such as unequal access to health care. Writing about this study, Benjamin (2019a) has further argued that while Obermeyer's analysis identified a harmful bias, it also points to the limits of solely targeting bias in analyses of individual algorithmic harms rather than focusing on the broader structural inequity that is produced by institutions within which such technologies are created and valued.

Using the concept of “coded exposure” Benjamin (2019b) has similarly suggested that facial recognition technologies produce distinct experiences of visibility that offer white people the privilege of privacy and reduced unwanted exposure to carceral surveilling institutions, such as in the context of facial recognition systems used for predictive policing and asylum and immigration. Benjamin problematizes efforts to develop more inclusive representative algorithms that are better able to recognize and distinguish non-white faces by arguing that as long as these technologies are deployed for carceral purposes, doing so will further forcibly expose BIPOC to government sanctioned containment and exploitation. This is especially so given the carceral expansion of the use of such technologies far beyond their original locations of prisons and borders into public spaces and private homes. Even technologies that are developed to redress or overcome racial biases in datasets and analytical processes—what Benjamin terms “technological beneficence”—end up reproducing or deepening discrimination because they are based on narrowly defined idea of fairness as a technical problem that is produced by individual intentions to do good. The aim to create race neutral technologies assumes that racism only exists in the meaning and tasks given to data and technologies by humans and can thus be prevented through better data and design (e.g., less racist engineers/companies). Such approaches are doomed to fail as they ignore neoliberal political economy and capitalist relations that underpin the development and deployment of AI-based technologies and sustain relations of white supremacy (Benjamin, 2019b). Benjamin and other critical race science, technology, and society (STS) scholars thus advocate for a refusal of technological solutionism that is rooted in abolitionist and solidarity politics with a focus on understanding and exposing intersecting algorithmic harms in order to imagine more liberating and equitable alternatives (Benjamin et al., 2019).

### Critical feminist studies

The implications of feminist analyses for understanding ageism in relation to the use of surveillance technology in nursing homes extend beyond comparative insights about people with different gender identities. Comparative insights reveal, for example, that women compared with men experience more data privacy risks and concerns, which are explained by differential experiences with and perceptions of sexual data leakage (Lageson et al., 2019), exploitation, and other online abuses (i.e., sexual harassment, doxing, stalking) (Bartel Sheehan, 1999; Messing et al., 2020). These explanations based on differential vulnerabilities to violence are worthwhile pursuits. They may be particularly worthwhile for aging studies considering gendered and racialized care labor and the fact that the number of men to women sharply declines at older ages, leaving far more women in the 85+ age category and short on care resources. But, as Hoffmann (2021) explains, “recognizing, visualizing, and caring for difference is ultimately insufficient if it leaves dominant logics and structures intact…” (p. 2). She recommends refusal as a universal feminist value—one of “commitment to declining the dominant social, political, or economic terms on offer” that here includes rejection of “the current terms of inclusion,” refusal of “empty calls for fairness and inclusion,” and of hollow, unmoved nods to difference and resistance (Hoffmann, 2021, p. 2–3). As Garcia et al. (2020) elaborate, “critical refusal is a generative concept for challenging harmful data practices, while simultaneously negotiating and developing alternative actions” (p. 2). This includes a consideration of “when to stop collecting data that does not support the rights of communities and when to stop building systems that introduce disproportionate risk and harm” (p. 2). Such a stance is uneasy with solutions of inclusive design as it is concerned with the ways in which enhanced visibility through data also intensifies vulnerability (Hoffmann, 2021). Feminist critical scholarship thus unveils the power concealed within data science practices and discourses. Creators of the Feminist Data Manifest-No articulate the value of practicing refusal (Cifor et al., 2019) with a bold and clear-eyed view that data science's diversity and inclusion are, in fact, roadblocks to liberation from the harms of social hierarchy.

## How would engagement with this critical scholarship direct our research on nursing homes?

This critical work has in common a refusal to accept technocratic solutions that lack evidence of efficacy and assessment of intersecting structural harms imposed on marginalized groups. What can thinking with insights from these fields addressing algorithmic harms do for gerontology? The concept of an interventional approach from disability studies (Davis, 2003; Hamraie and Fritsch, 2019; Williams, 2019; Shew, 2020) is already partially embedded in the STS-oriented subfield of socio-gerontechnology where a critique of an “interventionist logic” that dominates gerontechnology has been developed (Neven and Peine, 2017; Peine and Neven, 2018). By naming the interventionist logic, Neven and Peine (2017) and Peine and Neven (2018) make explicit some theoretical assumptions that have long been implicit in the design and research of technologies for older adults. They explain how gerontechnology has been guided by this logic in which aging is conceptualized as a target for biomedical and technological interventions—a set of problems to be solved. In a linear manner, a problem is identified, a user imagined, a technology designed to address it, which is then implemented with real older adults who must match the imagined user profile and use the technology in the way prescribed or scripted. Its impact is then evaluated using pre-defined outcomes. They argue that in fact socio-technical relations and practices are circular: aging, old age, older adults and technology are co-constituted (Peine and Neven, 2021). This STS-aligned intervention on the interventionist logic represents a welcome lens because, with some exceptions, gerontechnology has been active or complicit in framing older adults as in need of technological intervention in the form of datification for risk assessment. Research drawing on critical gerontological analysis of the tenacious normativity in models of aging (Katz and Marshall, 2004; Katz and Calasanti, 2015) has interrogated the interventionist logic and its implications. It has outlined dimensions of power relations involved in various types of data practices (Dalmer et al., 2022), their normalizing function in the service of biomedicine (Joyce and Loe, 2010; Katz and Marshall, 2018), and the meaning of the datafication of care in the context of austerity (Joyce and Loe, 2010; Mort et al., 2013; Sousa, 2013). Below we outline four broad moves for generative engagement with critical disability, race and feminist scholarship on data technologies to further expand the interdisciplinary lens of critical gerontology and enrich our analyses of surveillance technology, AI, and algorithmically mediated decision making in elder care. We provide some of many possible responses to the question, how would engagement with this critical scholarship direct our research on nursing homes?

### We would take surveillance and control seriously

Higgs and Gilleard (2021) describe an asymmetrical bifurcation in which technologies for older adults are often directed “toward those least able to exercise control but who are most susceptible to being controlled by others” (p. 1). Surveillance is particularly common in nursing homes and assisted living given that these are “total institutions” where the daily life and activities of residents are scheduled and monitored by care workers (Frik et al., 2019). While there is limited research on the impact of surveillance technologies for COVID-19 on residents, research pre-COVID-19 on these technologies suggests that these are most often used to monitor residents' behavior in ways that reinforce differential power relations between them and care workers and administration. For example, it is assumed that surveillance technologies used to locate and track the whereabouts of people living with dementia enhance their autonomy, independence, and safety by enabling a greater freedom of movement and the power to do what one wants (Wigg, 2010; Niemeijer et al., 2015; Ienca et al., 2018). Yet, there is little evidence of this benefit being achieved, and research suggests that these technologies may sometimes be used to supplement physical and environmental restraints (e.g., locking doors at night, bed restraints) (Wigg, 2010; Zwijsen et al., 2012; Timmons et al., 2019). The surveillance and confinement of residents through the physical environment is defended as necessary to protect the physical safety of “wandering” or “exit seeking” residents who are deemed as being so mobile that they interfere with provision of care or are in danger of getting lost (Wigg, 2010; Tufford et al., 2017; Steele et al., 2020; Graham, 2021). This is despite the fact that some people living with dementia perceive their “wandering” as meaningful and enjoyable (Adekoya and Guse, 2019) and would prefer to have freedom of movement and access to the outdoors (Steele et al., 2020). There is also ample research that demonstrates the negative impact of confinement on older adults, workers, and others, including confinement related to COVID-19 (Borges-Machado et al., 2020; Steele et al., 2020; Graham, 2021). While surveillance technology can be used on an as needed basis to locate an individual who becomes lost or to enable supervised movement (e.g., escorting the individual; Wigg, 2010), a more common finding is that surveillance is more widespread and used to supplement rather than to replace confinement and use of restraints (Grigorovich et al., 2021).

Assessment of risk through automated algorithmic decision making in resource-limited care environments is particularly valued as a strategy to reduce reliance on, or time spent on in-person monitoring (Grigorovich and Kontos, 2020; Ho, 2020). In this context, risk is imagined as a neutral and objective entity that is waiting to be found, rather than understanding the data collected and determined to signify risk as a structuring force. Identification of risk in this context can be based on ageist, ableist and other stigmatizing norms. Consider the meaning and impact of aggressive actions and movements by people living with dementia that are assumed to be disease-driven behavioral symptoms (Grigorovich et al., 2019; Grigorovich and Kontos, 2020). These symptoms are described as a major threat to quality of life and delivery of care in nursing homes, and there is growing interest in the development of predictive algorithms and surveillance technologies “to more immediately and accurately diagnose and manage” them (Husebo et al., 2020, p. 8). While such technologies may be accurate in distinguishing between individuals who are or are not aggressive, they cannot identify those who will actually exhibit aggression in the future. Moreover, such approaches are driven by the assumption that aggression is primarily the result of abnormal physiological or biochemical processes in the brain, and that identifying individual predictors or “triggers” of it can enhance providers' abilities to promptly defuse and redirect it. What is left unaddressed in such technological solutions are the structural influences of aggression (e.g., overcrowding, boredom, low provider-to-resident ratios, limited provider autonomy, and heavy workloads), which cannot be measured or addressed using surveillance technology or automated algorithmic decision-making. As we discuss further in our discussion of opportunity costs, the use of surveillance technologies in this way could distract from these structural problems and retrench professional and policy strategies that focus on restricting the freedom of individuals using behavioral modification, drugs, or other restraints with the intent to protect others from harm.

Surveillance technologies thus pose a disproportionate risk to residents' self-determination by characterizing them as threatening to others and by extending the power of care workers and administration to control their lives (Kenner, 2008; Shore, 2021). This is especially likely when algorithmic predictions counter the preference of a resident or conflict with their own assessment of risk associated with a given behavior (Ho, 2020). In this situation, both automation bias and the reinforcement of recommendations enabled through surveillance technologies could intensify unequal power dynamics between older adults and familial and professional caregivers (Ho, 2020).

For example, it is possible that the use of surveillance technologies for digital contact tracing in the context of the COVID-19 pandemic could contribute to greater use of physical or environment restraints in an effort to restrict the movements of infected residents who are determined unable to remember to isolate and/or use personal protective equipment (PPE). This is particularly concerning given the severe COVID-19-related restrictions that have already been imposed on residents, including their confinement to their room, chronic lockdowns and various other restrictions (Borges-Machado et al., 2020; Heckman et al., 2021). There has been a documented rise in already high use of antipsychotics in nursing homes in the context of COVID-19 (Campitelli et al., 2021), some of which may be related to the justification of using chemical restraints for infection control purposes (Keng et al., 2020). It is reasonable to expect the same rise in restraint as a consequence of digital contact tracing. Understaffing and lack of human resources in this sector drive interest in the development of predictive algorithms for automated decision support with regard to risk of falls or functional decline (Wojtusiak et al., 2021; von Gerich et al., 2022). Harms may be exacerbated if these technologies are also used to inform care planning, including decisions regarding the amount of care received or selection for intervention. Yet, such algorithmic harms are largely unrecognized in gerontechnology research or in tech research more broadly, which tends to reductively focus on informational threats to individual privacy that result from data shared from these technologies with stakeholders outside the circle of care (for example see: Oude Weernink et al., 2018; Bourbonnais et al., 2019).

Nursing home residents' right to privacy is recognized within both U.S. and Canadian law and considered important for residents' quality of life (Koren, 2010; Burack et al., 2012). The promotion of resident privacy as part of the nursing home culture change movement, which, since the 1980s, has motivated models of nursing home reform in both countries and beyond; culture change movements promote the creation of more home-like living environments with care worker and resident empowerment at their core (Koren, 2010; Dupuis et al., 2016). This includes supporting both decisional and physical forms of privacy through environmental design (e.g., private rooms) and residents' and workers' negotiation of schedules, activities, and care tasks (Grabowski, 2021). Privacy is also consistently reported as being important to older adults across a variety of living environments, including nursing homes in relation to the use of surveillance technologies (Garg et al., 2014; Berridge, 2016; Moyle et al., 2020). The potential threat to privacy posed by surveillance technologies may be even more salient for marginalized older adults, including racialized, poor, disabled, or LGBTQ+ older adults. While research in this area is very limited, a recent Australian study of social robots to combat loneliness found that older LGBTIQ+ adults living in the community identified fears of discrimination based on gender identity and sexual orientation and prioritized privacy in relation to surveillance technologies (Poulsen et al., 2020). Engagement with critical scholarship on information and data technologies would lead us to call out the meaning of privacy to older adults' lives and to further conversations regarding whether and how these can be upheld with the use of surveillance technologies. It would move us to take seriously the implications of privacy for non-conformity, freedom of association with others, and self-determination also with attentiveness to marginalized older adults who may have unique experiences and concerns.

Privacy implications are particularly significant in the context of nursing homes as there is ample research to suggest that residents' rights to different types of privacy (Koops et al., 2016) are already rarely upheld in practice. For example, the panopticon open-concept and congregate living design of many offers little opportunity for retreat from other people, other than one's room, which is most often shared with at least one other resident. Even when residents have private rooms, these rarely have door locks, and care workers at times open closed doors without knocking and bar residents from entering their own rooms during the daytime (Tufford et al., 2017). Violation of privacy in these ways is considered acceptable and is consistent with their duty of care to protect residents from harm. Nursing home culture change tenets would position this as the exception to the rule; however, research has found great variability in adoption of culture change practices. Higher rates of adoption are associated with lower proportions of Medicaid residents (Chisholm et al., 2018). The benefits of life in a nursing home that has adopted culture change practices, including respect for privacy, are thus largely restricted to more affluent and white residents. Within this privacy-depleted context where privacy still matters, it is especially important to prevent additional threats to privacy. Even if older adults do not express a desire for privacy or lack awareness of the impact of surveillance technologies on privacy, its protection is nonetheless important to avoid stigmatization and unnecessary restrictions on freedom.

#### This includes surveillance and control over workers

The expansion of surveillance technologies for contact tracing in nursing homes also has implications for the ways care workers are directed, evaluated, and disciplined. As with residents, interest in these technologies for monitoring workers in nursing homes pre-dates the pandemic and is part of a broader phenomenon of algorithmic management across sectors such as retail, manufacturing, banking, hotels, and call centers (Grigorovich and Kontos, 2020; Wood, 2021). Pre-COVID-19, care workers expressed worry that technologies used to monitor residents could also be used to sanction workers if used to monitor their performance (Bowen et al., 2013; Hall et al., 2019) and that this surveillance could undermine care relationships (Berridge et al., 2019b). Currently, surveillance technologies enhance classification and identification of patterns of movements and interactions with residents, which are interpreted by human managers. However, given the use of similar systems to automate performance evaluation in other sectors, it is likely that these technologies will be further developed to enable similar forms of automated decision-making.

These technologies are imagined as benefiting nursing home workers by enhancing their efficiency and optimizing care processes, including alleviating the burden of administrative tasks and increasing the time they can spend on care and social interaction (Oude Weernink et al., 2018). Much of the research on the impact of these types of technologies on workers has focused on the introduction of digital hailing and platform labor applications for services provided in private homes (Glaser, 2021; McDonald et al., 2021; Williams et al., 2021), including Electronic Visit Verification (EVV) for Medicaid home-based personal assistance services and home health care programs in the U.S. (Gallopyn and Iezzoni, 2020; Mateescu, 2021). Extensively critiqued by disabled activists who formed the Stop EVV campaign in 2017, this surveillance technology involves oversight of care workers (e.g., real-time tracking of arrival and departure times, locations, and activities), and by extension, low-income disabled beneficiaries living in the community. This requirement that is intended to gain efficiency and accountability has now been identified as another state practice that specifically penalizes a significant proportion of the working poor (Eubanks and Mateescu, 2021). Both beneficiaries and workers lack protections to restrict the scope of collected and inferred data to ensure that they are not at increased lifetime risk of punitive interventions from agencies making future use of the data produced about them that may be used to train predictive or other evaluative systems (Hopkins, 2018; Metcalf, 2018; Scalia, 2019). This is an example of how surveillance technologies create vulnerabilities that are not remedied by creating less biased algorithms. Moreover, given the racial dynamics of this workforce (Sloane et al., 2021), such technologies may reinforce racist labor inequalities by extending the power of employers to control low-wage BIPOC workers by undermining their labor rights and protections (van Doorn, 2017; Glaser, 2021; Mateescu, 2021; Williams et al., 2021).

### We would incorporate refusal into our analytical lens

Within the bias and fairness discourse in data science, data inclusion is positioned as an imperative, and so it is within emerging work on digital ageism. Inclusion promises to resolve the problem, defined in this case as age discrimination, through and within surveillance technologies. Expertise cannot, under this approach, fall to older adults or care workers who, along with long-term care systems, become dependent on technological interventions. There is also an inconsistency with which the value of expertise is applied. Ageism is readily called out in projections of older adults as technological laggards or as technologically incompetent. Their lack of positioning as experts in their own technological experiences is an acknowledged problem. At the same time, certain reasons that older adults provide through surveys for not adopting technologies like lack of interest or privacy concerns (Baig, 2021) tend to be downplayed (Berridge et al., 2022). Older adults' acts of resistance and rejection, along with reports of intentional non-adoption for reasons other than access or digital literacy barriers, are frequently overlooked in the framing of the problem (see Chu et al., 2022). For example, older adults may, in resisting a given technology, be taking a stance against the devaluation of cultural values like privacy (Barros Pena et al., 2021), but such studies that take seriously and engage the meanings of older adults' preferences and choices that are not tech-positive are quite rare. A contributing factor may be the successful rhetorical power of what Neven and Peine (2017) term the “aging-and-innovation discourse” that positions aging as a grave problem and legitimizes technology investment to solve it. The performative power of this discourse in which society, governments, and older people are all winners when technology is applied to the “problem” of aging (the “triple win”) is morally charged and thus difficult to argue with (Neven and Peine, 2017).

While enhancing autonomy is one of the most cited goals within gerontechnology, assumptions about older adults' values and about their capacities have served as justification to not involve or by-pass them in technology use decisions. A study in low-income senior independent living apartments of the long-term use of a sensor system that used algorithms to issue alerts when one deviates from their routine or stays in the bathroom “too long” identified multiple moments of boundary intrusion (Cohen, 2013), from adoption decision making through use over time (Berridge, 2016, 2017a,b). Techniques employed by building social workers and family members to convince residents (who were not living with dementia) included “revisiting” decisions not to adopt the sensor system, using moralizing discourse in which adoption was presented as the right thing to do and as indication that one wants to take care of oneself, issuing ultimatums (“it's this or a nursing home”), and bypassing the resident by bringing in a family member to decide, in conflict with the organization's own self-determination ethos and policy of independent living (Berridge, 2017a,b). Acceptance of these surveillance technologies was presented as the right option (“I say it's all up to you. How much you value yourself, how much you want to take care of yourself?”) and refusal was deemed “irrational thinking” (Berridge, 2017b, p. 82). Despite the heavy “selling” (social workers' term) of the subsidized system, only two percent of those targeted for use accepted it and 20% of those who had accepted it over the past 12 months had discontinued it. Among users, creative use of this system abounded (Berridge, 2017b). Rejection and what's termed “misuse” of technology by older adults is often dismissed as non-compliance, tech incompetence, or, as Neven (2015) articulates, “initial” vs. real resistance in the face of strong moral discourse embedded in technological innovation targeted on independent living. Using the lens of refusal from critical race and feminist scholarship (Benjamin, 2016; Hoffmann, 2021), we see that many residents in the sensor system study refused the limiting solutions on offer. A social worker who had emigrated from a Russian-speaking country described “socialist thinking” on the part of the significant population of residents from the same region who, across the board, declined offers of the system (Berridge et al., 2019a). Residents she worked with responded with questions like “Why should I have to depend on a piece of plastic for my life?” She recounted how a resident who was able to access in-home health aide services through Medicaid burst into her office and confronted her for the unfair treatment of her neighbor who, as the social worker understood but the resident did not, was near-poor but not yet Medicaid eligible and thus denied access to a home aide. These residents refused the technological solutions on offer and refused acceptance of the U.S.'s stringent means tested long-term care system as one that is just.

That sensor system's owning company has since clarified that they have moved from independent living into higher care settings (Corbyn, 2021). Barriers to refusal are far higher in the nursing home setting than in independent living for a few reasons, including age-graded algorithmic and data flow awareness (Gran et al., 2021). And while heavily regulated relative to other locations of long-term care, this is a location where ageism collides most directly with ideals of self-determination and person-centered care. Installation of sensors and other data-intensive technologies are more likely in all rooms as an institutional investment, for marketing purposes, or the default is an opt-out system of monitoring (Wetsman, 2020). As an example of this, a Canadian province recently invested in a private company that created an RTLS “to help grow its inventory and scale up to reach new markets” with the intent of implementing this system in long-term care homes to enhance safety and quality of care (Atlantic Canada Opportunities Agency, 2021). This demonstrates a growing public policy interest in Canada in implementing such systems in nursing homes as a long-term strategy for surveilling residents, workers, and visitors. Depending on one's location and access to funds, it can be very hard to find a facility, so moving into a nursing home without surveillance technology in use may not be a freely given choice. Objections, including refusals, that may be partially shaped by experiences of gender and sexuality, race, and socioeconomic status, will be important to take seriously when seeking to subject residents to technologies that collect and share private data (Cifor et al., 2019; Poulsen et al., 2020).

While roughly 30–50% of nursing home residents, in the Canada and the US respectively, are not living with dementia (CDC, 2019; CIHI, 2019), dementia heavily impacts residential life. Research has shown that people living with dementia often prefer to be more involved in decision-making than they are (Miller et al., 2016), and their preferences and concerns can go unrepresented (Whitlatch et al., 2005; Harman and Clare, 2006; de Boer et al., 2007; Menne and Whitlatch, 2007). Nudging, appealing to authority, incentivizing, and even deceiving and coercing people who lack decision making capacity in order to overcome their resistance to activities that are deemed helpful for their care are considered necessary practices (Nordgren, 2018). The ethical aspects and ramifications of deceiving people living with dementia in relation to surveillance technology use, from location or activity tracking to AI companions has proven of broad interest, perhaps because of its very sticky nature. Nordgren (2018) cautions that influencing the use of surveillance technologies with people living with dementia should be held to a more restrictive standard than those care practices deemed necessary to the survival, health and hygiene of individuals (e.g., requiring eating and bathing) because these technologies are supplemental and not necessary. They also raise the issue that decisions about tech use are subject to “technological ambitions, commercial opportunities and the wish among high-level decision-makers for cost-effectiveness in the use of limited health care resources” (Nordgren, 2018, p. 416). They recommend that decisions about what level of influence to exert over someone living with dementia be made on a case-by-case basis; however, this is a time-intensive task that nursing homes are ill-equipped to carry out. Deployment of adequate resources to provide person-centered care—arguably the missing piece that drives adoption of surveillance technologies in these settings in the first place—cannot logically be the solution. Alzheimer Europe (2010) published relevant ethics guidelines on surveillance and other types of technologies more than 10 years ago. While they may have a role in advancing the conversation, ethical guidelines which lack enforcement have little chance of making their way into on-the-ground decisions that impact nursing home residents' daily lives.

### We would understand and accommodate the limits of a consent model

Critical scholarship on information and data technologies focused on race, gender, and disability makes clear that when we talk about ethics we need to attend to power. How are surveillance technologies amplifying or addressing power imbalances at the structural and interpersonal levels? Hoffmann (2020a) reminds us that “doing ethics will mean attending to histories, dynamics, and relationships that exceed any given tool or technology” (p. 5) and that “ethical debate is not only about values, but about how we account for certain non-ideal facts about the world” (p. 6). Non-ideal facts include the fact that not everyone is capable of giving informed consent or opting out. Oppression is a non-ideal fact visible in structural ageism and ableism. Paternalism characterizes a lot of elder care but so does abandonment. Having limited options to get needed support is certainly disempowering. The point is, while the problem of surveillance technologies in elder care may get framed as a consent issue due to capacity limitations or one of respecting decisional autonomy, it is in fact a bigger problem of power. Following Viljoen (2021), it requires serious engagement with the social and historical context of nursing homes and of other manifestations of ageism and ableism. Informed consent often is not on the table in nursing home settings for a significant number of residents who either lack capacity to consent or are perceived to lack capacity. This environment and this population underscore the need, as Barocas and Nissenbaum (2014) have argued, to understand that informed consent is not the end game. Consenting processes are intended to enable individuals to protect their privacy, but to reduce the concept of privacy to control is to abandon additional questions about information flows that are disrupted or threatening to values that matter to people in this context (Nissenbaum, 2009). If the focus is on achieving consent from a resident or legal guardian, it is unlikely to also be on the potential harms of AI in nursing homes. Consent can serve, in this way, as a loophole to circumvent critical questions about what and whose values a given information flow protects or threatens (Barocas and Nissenbaum, 2014). It is a particularly problematic loophole where roughly half of a nursing home's residents' right to informed consent is ceded to their legal representative (Levy et al., 2018).

The nursing home context presents additional problems for relying on consent. Conversations and policies about consent often do not consider other actors, including workers and visitors, as interested parties in surveillance or data collection practices even when they become its subjects, inadvertently or not (Levy et al., 2018). Constrained choice or pressure to adapt to unfavorable circumstances (Zwijsen et al., 2011) are often-overlooked realities that complicate consent, as is the difficulty, distinct from comprehension, of appreciation for how a given technology will impact someone's daily life (Halpern et al., 2019). Consent should be an ongoing process in which people can change their minds, but this is impractical in a facility with staff shortages where an investment in a given device or surveillance system has already been made.

### We would call out the opportunity costs

What are some of the opportunity costs of massive public and private investment in surveillance technologies in resource restricted care environments over attention to the structural causes of vulnerability? Nursing homes became early adopters of digital contact tracing *via* proximity tracing to mitigate COVID-19 (Laroche, 2020; Wetsman, 2020). Digital contact tracing was presented as a promising response to nursing home infections where nearly 10% of U.S. and nearly 8% of Canadian nursing home residents have died from COVID-19 (CIHI, 2021b; The COVID Tracking Project, 2022). Many of them did not have the ability to say goodbye to loved ones and many have died alone with inadequate staffing that predated the pandemic. Surveillance technologies were promoted to protect essential workers during insufficient access to the vaccine as recently as September of 2021 in one Canadian province despite persistent capping of paid sick days at 3 for COVID-19 (Government of Ontario, 2021; Ontario Health Coalition, 2022). Testing for the virus was slow to arrive to desperate facilities and was disastrous with faulty kits finally sent. Few states in the U.S. required ongoing testing of residents or care workers in the early months of the pandemic. Facilities in the US. and Canada needed, and many are still struggling, to get access to PPE and fast, reliable, frequent testing (Braun et al., 2020; Murray and Friedmann, 2020; Paulin, 2021; Osman and Woolf, 2022). They lack adequately paid workers who don't have to work other jobs that put them at greater infection risk, proper infection control measures, single-occupancy rooms or other resources to overcome logistical problems with quarantining, or resources to manage health conditions without transferring to hospitals that put residents at greater infection risk. The public is asked to imagine, what can technological innovation do for people at risk for COVID-19 in nursing homes? A critical analysis interrogates the disconnect between problem and solution. Our question should be, what are the consequences of being preoccupied with how surveillance technology can save them?

To what extent does the increasing demand for surveillance technologies in nursing homes enable the retreat of governments from care responsibilities? To what extent does it distract attention and resources from known problems, as defined by those most concerned and impacted? If stressors are perceived to be alleviated at a superficial level by these technologies, does that distract from systemic change and further entrench the status quo (Gary, 2021)? The inadequate long-term care workforce—especially the dementia workforce—is an urgent public health problem (Armstrong et al., 2020; APHA, 2021) that has not been met with a proactive policy response. According to the American Public Health Association, workforce challenges “include a limited public health infrastructure to support population-level action, inadequate public and private investments in LTC, disparate access to community-based services and supports, and weak dementia care quality assurance systems.” With COVID-19, the problem has intensified.

The American Health Care Association now reports that about three-fourths of nursing homes have seen a worsening workforce situation during the pandemic, and 94% report a recent staffing shortage (AHCA/NCAL, 2020). A recent study of nearly all U.S. nursing homes found a mean annual turnover rate for certified nursing assistants of 129.1% (Gandhi et al., 2021). Low retention of nurse aides has been linked to poor quality indicators in nursing homes (Castle et al., 2020). The field in the U.S. is overrepresented by women who make up 92% of the nursing aide workforce, and BIPOC workers (57%), with 37% of the total workforce identifying as Black or African American (PHI, 2019), most often working under the supervision of white managers (Sloane et al., 2021). These workers are undervalued in long-term care systems (Mauldin, 2019; Sloane et al., 2021) where pervasive systemic and interpersonal racism harms workers and residents alike (Sloane et al., 2021). While average wages are higher in Canada than in the U.S., in both countries, personal care/nursing aides in nursing homes are poorly remunerated (PHI, 2019; Estabrooks et al., 2020). The needed inputs to stabilize the workforce have been studied over decades and research-based recommendations are laid out clearly (Daly and Szebehely, 2012; Braedley et al., 2017; Scales, 2021, 2022), yet as with other public health problems that have seen surveillance technologies and other AI-based proposed solutions to inadequate public investment, technological fixes are holding court. The question becomes, why are the solutions that get traction serving shareholders and not public health?

It is possible to stop at this level of analysis of what is wrong with nursing homes and to call for policy change to enhance their resources to better train and compensate workers. Yet the critique we have laid out thus far centers on ways in which surveillance technologies would further harm residents and workers, further surveil, normalize, and manage. In other words, further institutionalize them. These surveillance-mediated practices would entrench the problems of the nursing home, making a bad situation worse. And that situation is already worse for BIPOC, low-income, LGBTQ+, and people living with dementia because they are rendered even more vulnerable by nursing homes themselves. As a result of systemic racism, care quality and outcomes are worse in facilities that house primarily Black or Hispanic residents (Mor et al., 2004; Smith et al., 2008; Fennell et al., 2010; Cassie and Cassie, 2013; Li et al., 2015; Mack et al., 2018). It is also documented that many LGBTQ+ older adults are terrified of the notion of moving into a nursing home due to awareness that they may be in greater physical or emotional danger or forced back into the closet (Singleton, 2018; Wilson et al., 2018; Knochel and Flunker, 2021). Receiving care in nursing homes amplifies the dangers to them of ageist transphobia and heteronormativity. And given that people living with dementia are often overridden in decision making when risks are concerned (Stevenson et al., 2019), the introduction of AI in this context could reinforce ableism and further reduce opportunities for self-determination.

To ground analyses of possible algorithmic harms in their context (Hoffmann, 2018) requires that we examine the problem of the nursing home. A critique of the digital push for its power to depoliticize problems focuses us on the political nature of the high rates of infection and deaths from COVID-19. Like prisons, jails, and Immigration and Customs Enforcement (ICE) detention facilities, nursing homes are by their congregate nature locations that cause vulnerability to viruses (Tremain, 2020). Plenty of ageist commentary sprung up that naturalized the deaths of older adults. But despite paltry supports for family caregivers, there was a rush in the beginning of the pandemic of family members discharging nursing home residents to protect their health. Ontario even made decision aids to enable residents and family members to decide about temporarily moving out of long-term care facilities due to COVID-19 (NIA, 2021). In some ways these efforts mirror movement by advocates to get others living in institutions out, including ICE detention centers and prisons.

The danger these institutions place residents and workers in during a pandemic is not the only thing they have in common. Disability studies scholar, Ben-Moshe (2013) proposes that “incarceration is understood as a continuum of carceral edifices, or as an institutional matrix in which disability is a core component” (p. 399). The carceral logic of nursing homes can be spotted in power relations, often embedded in nursing home policies that restrict residents' freedoms and opportunities in the name of management (Tremain, 2021). Whether one agrees with abolition arguments targeted on nursing homes or not, one cannot deny the cogent critiques that motivate it. Yet, abolition remains a very marginalized perspective within the aging space.

Disability studies writers have called gerontologists and aging advocates out for this failure to embrace abolition, stating that it is a problem of their (our) lack of political analysis of incarceration/institutions (Boodman, 2019; Tremain, 2021). We agree with Herron et al. (2021) that this has roots in ageism, and add the general failure in gerontology to meaningfully engage the ways in which racism—both historically and present day—is embedded in and enables exploitative long-term care systems (for exceptions see for example Sloane et al., 2021; Jenkins Morales et al., 2022; Robinson-Lane et al., 2022). Insights on abolition from critical race studies and critical disability studies could lead to questions such as how are surveillance technologies inserted into established power dynamics in nursing homes? Where is the locus of power in the use of these technologies to inform care practices? It does not reside with residents or workers, and so surveillance technologies may further entrench the institutionalization of older adults and exploitation of workers. It will be critical to understand and further articulate how surveillance technologies and their role in automated algorithmic decision-making are in conflict with both abolition and reform approaches like the culture change movement that seeks to deinstitutionalize the nursing home.

## The COVID-19 crisis and beyond

Interest in the development of surveillance technologies for nursing homes pre-dates COVID-19. While the implementation of these technologies had largely been on an individual basis (e.g., used with residents who are identified as being most at risk), there was also documented interest in the more ubiquitous use of surveillance technology with all residents to enhance organizational protection from risk and liability (e.g., prevention of injury to residents, defense against allegations of negligence) or as leading to cost savings (e.g., reduction in providers, monitoring provider performance). There remains however a striking lack of evidence to demonstrate that these technologies can predict adverse events and decline in older adults or that they enhance prevention and health promotion. Even in the absence of such evidence, these technologies are seen as valuable for reputation management; that is, for mitigating family members' potential concerns about residents or as protection against complaints and litigation regarding neglect or abuse (Hall et al., 2019). While older adults and workers were largely reluctant to adopt these technologies pre-COVID-19, it is possible that this may shift with more commonplace data collection about one's physical health, including questionnaires, temperature screenings, and COVID-19 tests. Continued use of pandemic tracking apps for general health monitoring and for other types of infectious disease is likely (Seberger and Patil, 2021). Massive public investment in these technologies for workplaces (Government of Ontario, 2021), including nursing homes (Atlantic Canada Opportunities Agency, 2021) and the expansion of public health surveillance systems based on this type of big data in anticipation of future outbreaks further suggests they will remain in use (Miller, 2021).

## Discussion: concluding suggestions for focusing our work

Ageism can be a causal factor as well as consequence of an algorithmic harm. Yet ageism is not centered in critical scholarship in fields that have produced pioneering work on the impacts and harms of AI and algorithmically mediated decision making, and gerontology has largely failed to engage that critical work. In this article, we show key ways that critical race, feminist, and disability scholarship and activism can be mobilized to illuminate algorithmic harms as they relate to the problem of ageism. Drawing from this literature, we have explored possible algorithmic harms associated with surveillance, power and control that may fall to older adults and care workers within the U.S. and Canadian nursing home contexts. These harms extend beyond consequences of bias and problems of negative or inadequate representation. We begin with nursing homes, though this work has implications for home care that is also in need of study.

Ageism was deeply ingrained in nursing homes prior to the introduction of surveillance technologies, and together with ableist and racist logics, has shaped the nursing home context and the political environment in which they operate (Boodman, 2019). In centering ageism in conversation about algorithmic harms in the nursing home, we aim to understand the structural power of surveillance technologies and algorithmically mediated decision making by exposing how they serve to further reinforce the interests of neoliberal care regimes and the tech industry. We describe just some of many potential generative paths for further study to understand how surveillance technologies can exacerbate, amplify, and reinforce ageist practices of the nursing home that create vulnerability and reflect disposability. Future work should explore additional areas of critical data studies, including Indigenous and queer data scholarship.

We close with summative thoughts on specific ways in which such engagement could help us to draw out the power of ageism as a rhetorical and analytical tool. First, the COVID-19 crisis makes evident that calling out ageism without calling out racism is a problem, as is considering the harms of surveillance technologies that fall to residents or workers in isolation from each other. Following Herron et al.'s (2021) recent critique of dominant discourses of ageism in general, there is a need to decenter whiteness in scholarship and public discourse on digital ageism by considering the “rights of older people and the value of care work together” (p. 196). This includes attending to the impact of surveillance technologies not only on racialized nursing home residents of all ages but also on the racialized workers who care for them. Engagement with the critical race scholarship on surveillance technology should lead us to ask questions about the impact of these technologies in nursing homes on workers- the majority of whom are BIPOC and immigrant women—working in an environment already characterized by “ambient criminalization” (Mateescu, 2021). v This further includes confronting the role of racism as desirable and profitable within the technocratic solutions sold by the “aging enterprise” (Estes, 1979). For example, public and private investment in surveillance technologies over structural reform could disproportionately expose racialized workers who are already inadequately valued and supported (Estabrooks et al., 2020; Scales, 2022) to stigma and may further undermine their labor rights. This would be particularly of concern within under-resourced nursing homes, where racialized older adults are more likely to live, and could in turn reinforce existing racial disparities in access, process, and outcomes of care for residents.

Taking our lead from Benjamin (Benjamin et al., 2019), we would critically interrogate surveillance technologies with awareness that innovation in the care of older adults does not necessarily represent progress toward more just systems of care or practices. After all, medicating residents into submissive subjects was once considered a progressive innovation that was felt to have solved the problems associated with using physical restraints. Examining these innovations with a critical lens on impact rather than intention (Eubanks, 2018), would shed much-needed light on the harmful side of surveillance technologies in the nursing home, including the ways in which ageism is perpetuated by them.

Engagement with the concept of refusal developed within critical race and feminist studies would mean asking where can people living in nursing homes find opportunities for refusal. It would prepare us to confront unviable contradictions, such as in claims that surveillance technologies are targeted on enhancing older adults' independence where its purpose may actually be safety and risk management that could serve to diminish opportunities for self-determination (Berridge, 2017a). With an understanding of the importance of non-conformity and self-determination for all people, we would take actions to protect the privacy of residents. And with attention to the economically vulnerable status of the majority of nursing home residents, we might ask, how is the best interest of the resident living with dementia assessed given the unbalanced power of countervailing interests?

For non-age studies fields, a focus on ageism and dementia in the nursing home context further illustrates the limits of consent and reliance on autonomy-based control criteria to achieve ethical technology use. In the instance of dementia, capacity to give, refuse, or withdraw consent decreases as one's condition progresses, which could be the very trigger for surveillance technologies (Ho, 2020).

It also suggests that we take one of the pillars of AI ethics—the principle of fairness—further, by asking, is it fair to ask those who are some of the least likely to have the knowledge and experience with data-intensive technologies (Frik et al., 2019) or algorithmic awareness (Gran et al., 2021) to weigh attendant risks to agree to be among the most data-surveilled? This question makes evident the need for more attention among gerontologists to regulation, absent protective regulation both for residents and workers who also tend to be digitally marginalized. As Stypińska (2021) points out, while there is active work on ethical AI regulations and guidelines, the interests of older adults as a collective are thus far unrepresented. More concern with regulation would move the conversation quickly from one of tech ethics and issues of consent to one of power, and specifically of evidence of ageism in protective regulations or lack thereof. What's currently getting attention are the harms to older adults of being screened out through or within AI (Whittaker et al., 2019), but how are people differentially screened into surveillance in a way that prioritizes profit?

Finally, writing to date on digital ageism has focused on addressing individual harms of AI bias and exclusions. To fully flesh out ageism embedded in AI practices, an analysis of the social harms of surveillance technologies and automated algorithmic decision-making in nursing homes is needed. Individual harms are experienced directly by persons and the accumulation of those harms entails collective harm (Smuha, 2021). Social harms extend beyond those harms done to individuals (i.e., the individual nursing home resident) or collectives (residents and workers) who are directly subjected to a given AI application (Smuha, 2021). Social harms associated with surveillance technologies used in nursing homes might accrue to the vast majority of the population—to younger disabled and non-disabled adults who don't reside or work in nursing homes through the foreclosure of future alternatives to institutional surveillance in elder care. The negative consequences of both structural and individual level ageism on older adults' health have been extensively documented (Chang et al., 2020). Because we all are continuously aging and most are living with the expectation to personally experience old age, the negative health effects of ageism accrue very broadly across time. The ageism embedded in nursing homes and the ways that ageism stands to be exacerbated by surveillance technologies deeply impacts one's ideas about old age, including their own, whether one expects to end up in such a facility themselves or not. As Rosales and Fernández-Ardèvol (2020) explain, “Ageism shapes both the image(s) that individuals have of themselves and the image(s) that society has of the different life stages.” The way nursing home residents and workers are treated both reifies and normalizes ideas about old age care, including the idea that one's own older person is not worth the financial cost of person-centered attentive care, but that one would need to be efficiently managed through automated algorithmic decision making. That is, the use of surveillance technologies has the potential to help shape all ages' ideas about their futures, and in turn shape feelings like fear, dread or hope. Social harm is that which entrenches an impoverished imaginary of how ways of relating to each other in spaces of elder care could be otherwise. Refusal as a value is not just a rejection but a claim to the capability to imagine and create new futures (Benjamin et al., 2019).

To the extent that the use of surveillance technologies in nursing homes extinguishes will toward new futures of care, it creates social harm. We echo disability scholar Eva Boodman, who explains the need for no less than a paradigm shift:

nursing homes and institutional environments for older adults have a role in normalizing the idea that our elders are a burden, are objects of medical technology...What this means is that to shift their normalizing power, we will have to target not just the institutions of prisons and nursing homes, but the sets of norms, values, and auxiliary structures that support them and that are continuous with them (Boodman, 2019, p. 16).

We can look to scholarship and activism that embodies a politics of refusal of technocapitalist practices that threaten to entrench aspects of institutionalization within nursing homes that have sparked broad demand for reform, with a minority looking to larger abolition movements. We hope that identifying areas for inquiry to begin to explore algorithmic harms and their animating force within the under-recognized setting of the nursing home will contribute to coalition building and broader efforts to trace, measure, and take action to prevent algorithmic harms across sectors and marginalized collectives.

## Data availability statement

The original contributions presented in the study are included in the article/supplementary material, further inquiries can be directed to the corresponding author.

## Author contributions

All authors listed have made a substantial, direct, and intellectual contribution to the work and approved it for publication.

## Funding

CB was supported by the National Institute on Aging (PI: Berridge, NIA K01AG062681).

## Conflict of interest

The authors declare that the research was conducted in the absence of any commercial or financial relationships that could be construed as a potential conflict of interest.

## Publisher's note

All claims expressed in this article are solely those of the authors and do not necessarily represent those of their affiliated organizations, or those of the publisher, the editors and the reviewers. Any product that may be evaluated in this article, or claim that may be made by its manufacturer, is not guaranteed or endorsed by the publisher.
